# Solvent Retention Capacity and Gluten Protein Composition of Durum Wheat Flour as Influenced by Drought and Heat Stress

**DOI:** 10.3390/plants10051000

**Published:** 2021-05-17

**Authors:** Maryke Labuschagne, Carlos Guzmán, Keneuoe Phakela, Barend Wentzel, Angeline van Biljon

**Affiliations:** 1 Department of Plant Sciences, University of the Free State, Bloemfontein 9300, South Africa; phakelakeneuoe@yahoo.com (K.P.); avbiljon@ufs.ac.za (A.v.B.); 2 Departamento de Genática, Escuela Técnica Superior de Ingeniería Agronómica y de Montes, Edificio Gregor Mendel, Campus de Rabanales, Universidad de Córdoba, CeiA3, 14071 Córdoba, Spain; carlos.guzman@uco.es; 3 Agricultural Research Council—Small Grains, Bethlehem 9700, South Africa; wentzelb@arc.agric.za

**Keywords:** abiotic stress, durum, glutenin, SE-HPLC, SRC, SIG

## Abstract

Drought and temperature stress can cause considerable gluten protein accumulation changes during grain-filling, resulting in variations in wheat quality. The contribution of functional polymeric components of flour to its overall functionality and quality can be measured using solvent retention capacity (SRC). The aim of this study was to determine the effect of moderate and severe drought and heat stress on SRC and swelling index of glutenin (SIG) in six durum wheat cultivars with the same glutenin subunit composition and its relation with gluten protein fractions from size exclusion high performance liquid chromatography. Distilled water, sodium carbonate and sucrose SRC reacted similarly to stress conditions, with moderate heat causing the lowest values. Lactic acid SRC and SIG reacted similarly, where severe heat stress highly significantly increased the values. SIG was significantly correlated with sodium dodecyl sulphate sedimentation (SDSS) and flour protein content (FPC) under all conditions. Lactic acid SRC was highly correlated with FPC under optimal and moderate heat stress and with SDSS under moderate drought and severe heat. SIG was negatively correlated with low molecular weight glutenins under optimal and drought conditions, and combined for all treatments. The relationship between SRC and gluten proteins was inconsistent under different stress conditions.

## 1. Introduction

The importance of wheat, compared to other cereals, lies in its unique physicochemical properties. When wheat flour/semolina is mixed with water, a part of the protein found in the grain of wheat will form the viscoelastic protein network called gluten, which gives dough its ability to trap gas and stretch, both aspects necessary to produce wheat based products. The gluten properties greatly influence end-use quality of durum wheat [1,2]. In durum wheat, gluten gives dough the necessary cohesiveness to be extruded to form different pasta shapes when dried. Gluten is the primary element of protein in wheat grain, and consists of monomeric gliadins and polymeric glutenins, and makes up about 80–85% of total wheat protein [3]. Glutenin proteins are further divided into low molecular weight (LMW) and high molecular weight (HMW) glutenins. Gliadins are the most abundant protein contained in the wheat seed. Both glutenins and gliadins contribute towards durum wheat quality and confer dough visco-elastic properties [4]. Size exclusion high performance liquid chromatography (SE-HPLC), has been widely used for analysing cereal proteins. Proteins are separated and assessed according to characteristics associated with molecular weight distribution. The technique is accurate, sensitive, reproducible and easily automated [5]. SE-HPLC has been used in wheat studies to evaluate bread and pasta making quality [6,7].

Temperature and drought are implicated in causing considerable changes in the accumulation of gluten proteins during the grain-filling period, which affect dough rheological characteristics, resulting in variations in durum wheat quality [8]. High temperatures alter flour and dough quality, which has been associated with increase in gliadin/glutenin ratios [9,10].

All rheological and baking tests, traditionally used for quality evaluation, measure the major functional flour components’ combined contribution [11]. Analysing each individual contribution of the different functional components can assist in the prediction of the overall flour functionality, and an improved understanding of the mechanisms for dough mixing and baking. To overcome the cost and expense of milling and baking hundreds of samples, two rapid predictive tests for end-use quality assessment were developed namely; the Solvent Retention Capacity (SRC) test and Swelling Index of Glutenin (SIG) test [12]. These tests were found to be very useful in the prediction of bread and soft wheat quality [13]. The contribution of functional polymeric components of flour to the overall flour functionality and quality of the end-product, can be measured using the SRC method. It measures the capacity of flour to retain a set of four solvents including distilled water, 50% sucrose, 5% sodium carbonate and 5% lactic acid. Each solvent will predict different functional polymeric components [11] including gluten characteristics, damaged starch resulting from the milling process, pentosans or arabinoxylans that originate from the bran and aleurone layer that significantly increases water holding capacity, and gliadin characteristics.

Sucrose SRC is associated with gliadin content and differentiates flours with different water-soluble pentosans. Sodium carbonate SRC is correlated with the levels and swelling of damaged starch, or solvent-accessible amylopectin in damaged starch, as well as pentosans in flour. Lactic acid SRC relates with gluten strength by swelling the glutenin subunits, directly affecting dough strength. Distilled water SRC is influenced by the gliadin, pentosan, damaged starch and gluten strength flour constituents, while distilled water SRC reflects the flour’s ability to hold water. All four SRC solvents were significantly correlated with an increase of damaged starch content [14,15,16].

Wheat gluten holds approximately 2.8 g of water for each gram of dry gluten, starch only holds 0.3–0.45 g of water per gram of dry starch, damaged starch is able to retain 1.5–2 g of water per gram of dry starch, whereas pasted starch has the capacity to retain up to 10 g of water per gram of dry starch. Solvent accessible pentosans can retain up to 10 g of water for each gram of dry pentosans [17]. It was reported [18] that for a typical wheat sample, water absorption contributed by gluten is 27%, the combined absorption for native and damaged starch is 34% and pentosans absorb 25% of the total water of the sample.

The swelling index of glutenin (SIG) method determines the glutenin’s swelling power to predict dough quality characteristics and end-use quality, especially those associated with dough strength and baking characteristics, and only requires 0.04 g flour. The SIG test can evaluate flour quality between varieties with a broad quality range [19] and also varieties with a narrow range in insoluble glutenin content. The swelling capacity of glutenin depends on swelling time and mixing intensity in non-reducing solvents such as sodium dodecyl sulphate (SDS), lactic acid or dilute acetic acid. Quality parameters that are directly related to dough strength parameters and highly correlated to the insoluble gluten fraction, will be higher correlated with SIG test values [20,21]. The SIG method is simple, quick and more consistent in predicting insoluble glutenin content as well as dough strength characteristics than the Zeleny and SDS sedimentation (SDSS) tests, with a smaller experimental error. The SIG method allows high throughput with high reproducibility [20,21].

SE-HPLC is a technique widely used in the separation of wheat proteins. The technique separates proteins according to molecular weight distribution [5] and studies using SE-HPLC indicated that quantity and molecular weight distribution of gluten proteins are important in durum dough strength and pasta making quality [9,22].

The aim of this study was to determine the effect of moderate and severe drought stress, and moderate and severe heat stress on the SRC and SIG test in durum wheat cultivars, and how this relates to gluten protein fractions obtained from SE-HPLC.

## 2. Results

Moderate drought caused the highest values in distilled water SRC and sodium carbonate SRC for four of the six cultivars (Table 1). In five of the cultivars, for distilled water SRC, and in all cultivars for sodium carbonate SRC and sucrose SRC, moderate heat stress caused the lowest values. In the case of sucrose SRC, the value of the optimal treatment was the highest for three of the cultivars, and severe heat for two of the cultivars. For lactic acid SRC and SIG, severe heat consistently caused the highest values in all cultivars. For lactic acid SRC, either severe drought (three cultivars) or the control (three cultivars) had the lowest values. For SDSS, moderate drought values were the lowest for four of the cultivars and were the highest under severe heat for three of the cultivars. The control treatment consistently had the lowest flour protein content (FPC) values, and for five of the six cultivars, severe drought caused the highest values. The SDS extractable HMW glutenins determined by SE-HPLC differed highly significantly between many of the treatments (Table 2).

For the SDS unextractable HMW glutenins, there did not seem to be a distinct pattern due to treatments. For the LMW glutenins, the control had the highest values in four of the cultivars. Although stress treatments generally reduced the values, there were no patterns in the reductions. For the gliadins, moderate and severe heat stress caused the lowest values in five of the cultivars. AG was the highest for the control treatment for four cultivars, while moderate or severe heat stress caused the lowest values in four cultivars. For large and total unextractable polymeric proteins there was no clear pattern of values due to stress treatments.

When the SRC mean values per treatment were compared (Table 3) moderate drought caused the highest value for distilled water and sodium carbonate SRC (significantly higher than all other treatments), while moderate heat caused the lowest values (significantly lower than all other treatments). For sucrose SRC, all values were significantly reduced due to stress treatments. Lactic acid SRC was the highest under severe heat (significantly higher than all other treatments). Likewise, SIG was also the highest under severe heat stress (significantly higher than all other treatments). Moderate and severe heat stress caused the highest SDSS values (significantly higher than the two drought stress values). FPC was the highest under severe drought (significantly higher than all other treatments) and the lowest under control conditions. Although the yield results are not included in this study, the overall mean for two seasons for optimal conditions was 8.12 t/ha, for moderate heat 7.83 t/ha, for moderate drought 4.86 t/ha, for severe drought 2.52 t/ha and for severe heat 1.69 t/ha, which shows an inverse relationship with FPC.

When the SDS extractable protein means for all cultivars were compared (Table 4), the values were the highest under severe heat for both HMW glutenins and gliadins (significantly higher than optimum conditions and severe drought for both fractions), and the lowest under control conditions (significantly lower than all treatments except for moderate drought). For the LMW glutenins, the trend was reversed with the highest value under optimum conditions (significantly higher than severe heat and moderate and severe drought) and the lowest under severe heat (significantly lower than optimum and moderate heat treatments). For the AG, the value was the highest under moderate heat (significantly higher than severe heat and moderate and severe drought) and the lowest under severe heat (significantly lower than all but the severe drought treatment).

For the SDS unextractable (Table 4) HMW proteins, severe drought caused the highest values (significantly higher only than severe heat) and severe heat the lowest value (significantly lower than all other treatments except severe drought). The LMW glutenin was the highest under optimal conditions (significantly higher than all treatments except moderate drought) and the lowest under severe drought. Gliadins were the lowest under moderate heat and the highest under moderate drought (no significant differences between treatments). AG was the highest under control conditions (significantly higher than moderate heat) and the lowest under moderate heat (significantly lower than under optimal and moderate drought conditions). Large unextractable polymeric proteins (LUPP) were the highest under severe drought (significantly higher than under moderate and severe heat, and moderate drought) and the lowest under severe heat (significantly lower than all but moderate drought), while total unextractable polymeric proteins (UPP) was the highest under optimal conditions (significantly higher than all other treatments) and the lowest under severe heat conditions (significantly lower than all other treatments).

### Correlations

Under optimal conditions, the distilled water SRC was highly positively correlated with the LUPP and UPP, while both lactic acid SRC and sodium carbonate SRC were highly correlated with the FPC. SIG was highly negatively correlated with unextractable LMW and highly positively with SDSS and FPC (Table 5). Under moderate drought conditions, there was a large increase in number of significant correlations. The sodium carbonate SRC was significantly negatively correlated with extractable gliadin, FPC and SDSS, and water SRC with SDSS, while lactic SRC and SIG were positively correlated with SDSS. Under severe drought stress distilled water, sodium carbonate, sucrose and lactic acid SRC were all highly negatively correlated with extractable HMW. The only significant positive correlation was SIG with SDSS. Under moderate heat lactic SRC and SIG were both highly significantly correlated with FPC (positive) and distilled water SRC (negative). Under severe heat conditions, both sucrose and distilled water SRC were significantly correlated with extractable LMW (positive) and unextractable LMW (negative). SDSS was significantly correlated with lactic acid SRC (positive) and both water and sodium carbonate SRC (negative).

## 3. Discussion

The SRC method is based on energetic thermodynamic polymer-solvent compatibility, contrasting with the rheological methods based on kinetics of dough development [11]. Due to the simplicity of the SRC method, variation in SRC results between laboratories and technicians are minor when compared to rheological and baking test results [14]. The role of each of the three solvents in addition to distilled water SRC is to enlarge the contribution of one functional flour component, compared with its contribution to swelling in water. Swelling in the respective solvents will be the result of the water that makes up the solvent, but will to a greater extent be due to the solvent with the highest compatibility with the respective flour polymers in which levels are increased [17].

For distilled water, sodium carbonate and sucrose SRC in the individual durum cultivars, moderate heat consistently (with one exception) caused the lowest values. When combined for all cultivars, moderate heat caused the lowest distilled water and sodium carbonate values (significantly lower than all other treatments) and sucrose SRC values (significantly lower than the control and severe drought). These two solvents and distilled water SRC showed similar responses in the different cultivars and combined for all cultivars. This indicates that moderate heat stress caused a reduction in SRC when these specific solvents were used. As all four SRC solvents are water-based, their values as a measurement of individual polymeric component activity could be related [10]. Yet sucrose and sodium carbonate SRC are generally associated with gliadins, damaged starch and pentosans, while distilled water SRC is associated with gliadins and gluten strength, and pentosans and damaged starch [14,15,16]. SDS extractable gliadins were increased significantly due to moderate heat, while SDS unextractable gliadins were reduced, but not significantly, so this effect could not have been due to the gliadins. UPP was significantly reduced due to moderate heat stress, so this could have been a contributing factor. In this study white flour was used, which has reduced the effects of pentosans significantly. Damaged starch and pentosans were not measured in this study, which would have made a contribution to SRC. SRC was reported to be minimally affected by heat and drought stress [23].

For lactic acid SRC and SIG, in the individual cultivars, severe heat consistently caused the highest values (significantly higher than all other values), while for lactic acid SRC either optimal or severe drought conditions caused the lowest values, and for SIG either the control or moderate drought caused the lowest values. Heat and drought stress was reported to affect SIG test values, where drought stress caused an increase, and heat stress a decrease [23]. This was contrary to what was seen in the current study. It was found that the environmental effects were higher than genotype effects on SRC values in hard red spring varieties [24]. There were differences between cultivars in the current study, but stress treatments largely affected the values, showing large environmental effects on the SRC values.

The SDS extractable HMW for individual cultivars were consistently the lowest under optimal conditions (with one exception), and were generally the highest under either moderate or severe heat. With the exception of this fraction, there was no consistent pattern in how cultivars reacted to stress conditions. This emphasizes the effect of cultivar in response to stress conditions, and that effects cannot be generalized for all cultivars. When the average of the six cultivars was used for analyses, there was a significant increase in SDS extractable HMW and gliadin due to stress (with the exception of moderate drought for the gliadins). LMW and AG were significantly reduced from optimal conditions under severe heat, and under moderate and severe drought. The SDS unextractable HMW was significantly decreased under severe heat compared to optimal conditions, which was the opposite of the SDS extractable HMW. The unextractable LMW reacted similarly to extractable LMW, in that there was a significant reduction under all conditions except for severe drought compared to the optimum. There were no significant differences in the unextractable gliadins due to stress treatments. Unextractable AG was significantly reduced under all conditions, excluding moderate drought. LUPP was significantly reduced from optimal conditions under severe heat and moderate drought. UPP was significantly reduced only under severe heat and severe drought. This data supports previous reports that temperatures above 35 °C caused a reduction in larger polymeric proteins and had a dough weakening effect [8,9]. Contrary to this, an increase in HMW glutenins was seen at 37 °C [23], which was also the case in the current study, but only for the SDS extractable HMW which were increased due to both heat and drought stress.

The SDS sedimentation test in combination with grain protein was found to be adequate to screen durum wheat for gluten strength and spaghetti cooking quality [25]. In this study, SIG and SDSS were consistently highly significantly correlated, irrespective of the treatments they were subjected to. SIG was also significantly positively correlated with FPC under optimal and moderate heat conditions and across all treatments. This confirms that the SIG method measures the swelling power of glutenin, and can be used to predict dough quality characteristics associated with dough strength [19]. SIG was significantly negatively correlated with unextractable LMW under optimal conditions, and extractable LMW glutenin under moderate and severe drought stress. Significant correlations of SIG values with the insoluble glutenin test and SDSS test were reported previously [19]. The SIG values obtained in their study were significantly better correlated with insoluble gluten content than SDSS and Zeleny sedimentation tests, indicating that the SIG test is a better indicator of insoluble glutenin content. Subunits coded by *Glu-A3* loci, *Glu-B3* and *Glu-D3* significantly affected gluten strength as measured by SIG, and the highest correlations with gluten strength were expressed with 2 *, 17+18, 5+10, *Glu-A3*, *Glu-B3g* and *Glu-D3b* subunit combinations [26]. These results are contradictory to another study [20], which reported that the SIG measures insoluble glutenin mainly consisting of HMW-GS located on the *Glu-1* loci. In the current study, using durum wheat, all cultivars had only *Glu-1* loci subunits 7 + 8, coded by *Glu-B1*. The SIG was highly negatively correlated with the LMW glutenins under optimal, and moderate and severe heat conditions, but there was no relationship with the HMW glutenins.

Lactic acid SRC was highly significantly correlated with FPC under optimal and moderate heat conditions, and with SDSS under moderate drought and severe heat. Lactic acid SRC was also correlated with LUPP under moderate heat conditions. This supports the notion that lactic acid SRC relates to gluten strength by swelling the glutenin subunits, directly affecting dough strength [16]. It was reported [17] that flour proteins, but especially the glutenins, contributed to lactic acid SRC values.

Sodium carbonate SRCand sucrose SRC were significantly negatively correlated with SDSS under moderate and severe drought and moderate heat. Distilled water SRC was significantly correlated with LUPP and UPP under optimal conditions, but was significantly negatively correlated with extractable HMW under severe drought conditions. Low correlations of SDS extractable protein with SRC values and bread making quality parameters were reported using SE-HPLC [27]. However, the SDS unextractable proteins, especially the HMW polymeric protein fraction, were found to be highly correlated with distilled water SRC, sodium carbonate SRC and lactic acid SRC values and less significantly correlated with sucrose SRC. In the current study, there were only a few significant positive correlations of SRC with unextractable proteins (sodium carbonate SRC with unextractable HMW, and lactic SRC with LUPP, both under moderate drought, and distilled water SRC with LUPP).

The LMW polymeric protein fraction was previously reported to be highly correlated with lactic acid SRC, due to the exaggerated swelling action of the glutenin network in lactic acid. The distilled water SRC solvent was also reported to be significantly positively correlated with both the HMW polymeric protein fraction and the gliadin fraction [11]. This was not the case in the current study, where no significant association of distilled water SRC with extractable or unextractable LMW was seen.

In conclusion, moderate heat caused significantly lower distilled water and sodium carbonate SRC values than all other treatments and sucrose SRC values significantly lower than control and severe drought conditions. SDS extractable gliadins were increased significantly due to moderate heat and UPP was significantly reduced due to moderate heat stress. Lactic acid SRC and SIG had a similar response to severe heat stress, with highly significantly increased values. SIG was significantly correlated with SDSS and FPC under all conditions. Lactic acid SRC was also highly correlated with FPC under optimal and moderate heat stress and with SDSS under moderate drought and severe heat. This study showed that lactic acid SRC and SIG can be used as predictors of end-use quality under optimal and stress conditions.

## 4. Materials and Methods

Six durum wheat cultivars (MexicaliC75, YavarosC79, AltarC84, AtilC2000, JupareC2001 and CirnoC2008) from the International Maize and Wheat Improvement Center (CIMMYT) durum wheat breeding program were used. They were selected based on the same HMW and LMW glutenin composition as determined by SDS-polyacrylamide gel electrophoresis (SDS-PAGE) (Table 6). This was done to minimize the effect of glutenin variation in relation to SRC and SIG.

### 4.1. Trial Designs and Treatments

The trial was laid out as a randomized complete block design, with three replications. The trial was conducted in six different growing conditions: full drip irrigation (optimum conditions), reduced irrigation or moderate drought stress, severe drought stress, moderate heat stress and severe heat stress. The genotypes were sown at Ciudad Obregon Sonora, in Northwest Mexico in two consecutive growing seasons. The seeds were sown in November for all the treatments except for moderate heat stress (planted in January) and severe heat stress (planted in February). During grain filling, the maximum temperatures varied between 31 °C and 32 °C in March and April for all the treatments, excluding genotypes under severe heat stress, where temperatures were around 35–36 °C in May (Table 7).

All the trials were grown under full irrigation (>500 mm) except for moderate drought stress (300 mm) and severe drought stress (180 mm). The data obtained from Ciudad Obregon metrological station showed an almost total lack of rainfall during the wheat growing season. Under moderate and severe drought stress, the plants were subjected to drought stress from stem elongation until physiological maturity. Soil characteristics are given in Table 8.

Under heat stress conditions, the plants experienced higher temperatures from shoot elongation until seed ripening. The N fertilization was optimized for each environment. At sowing, all the trials received nitrogen (N) application of 50 kg/ha and 150 additional units of N at tillering in all treatments, except for severe drought stress, which received only 50 additional units of N. The reason for this was that under drought stress the yield potential is reduced significantly and the plants do not need that much nitrogen to yield according to the environmental potential.

Required agronomic practices were applied and weeds were removed. When the plants reached physiological maturity, the whole plot was harvested and 1 kg of seed obtained from two replications of each genotype was used for quality analysis. Grain samples were conditioned to 16% moisture content, milled into flour using a Brabender Quadrumat Jr. (C.W. Brabender OHG, Duisburg, Germany).

### 4.2. Flour Protein Content and SDS Sedimentation

The protein and moisture content of flour was determined using near-infrared spectroscopy (NIR Systems 6500, Foss Denmark) according to American Association of Cereal Chemists (AACC) methods 39–10 and 46–11A [30]. FPC values were expressed based on 12.5% moisture basis. SDSS was measured according to AACC approved method 56–70 [31].

### 4.3. Solvent Retention Capacity (AM56-11 Modified Method)

A modified protocol [15] of the Approved Method 56-11 [30] was followed using four water based solvents. Flour samples of 0.3 g, with a moisture content of 14%, were weighed into a 2.0 mL centrifuge tube of known weight. Four SRC solvents were independently prepared using distilled water and used to obtain four SRC values. The four solvents included the following: distilled water, a 5% (*v*/*v*) lactic acid solution, a 5% (*w*/*v*) sodium carbonate solution and a 50% (*w*/*v*) sucrose solution.

For the preparation of the 5% (*v*/*v*) lactic acid solution, the actual assay value on the reagent bottle was used to calculate the appropriate amount of lactic acid to be diluted for the solution. To avoid kinetic effects on flour solvation and swelling, the solvents used in the SRC method are each used in a fivefold ratio to flour, 1.5 mL of the solvent to 0.3 g of flour according to the modified protocol of the AM 56-11.02 [30].

Freshly prepared lactic acid solution was used. The 50% (*w*/*v*) sodium carbonate solution was prepared a day before use to allow the sodium carbonate to dissolve completely. The sodium carbonate concentration was calculated on a weight basis instead of volume [11].

After 1.5 mL of the appropriate solvent was added to the tubes, it was mixed in a vortex until suspended for 10 s. The vortexed tubes were immediately placed in a thermomixer block (Thermomixer^®^, Eppendorf AG, Hamburg, Germany) to shake at 1400 rpm for 5 min at 25 °C. The flour solvent suspensions were centrifuged at exactly 4000× *g* for 2 min. After centrifuging, the supernatant liquid was discarded and the tube drained at room temperature for 10 min. The lid of the tube was dried with tissue paper. The tube weight, including that of the lid and gel, was determined and the SRC calculated as the sum of the tube and gel weight less the original empty tube weight divided by the original flour weight, thus the SRC value was calculated as a percentage of flour weight on a 14% moisture basis.

### 4.4. Swelling Index of Glutenin

The SIG values were determined using the method of Wang and Kovacs [19]. A flour sample of 0.04 g with a 14% moisture content was weighed into a 2.0 mL centrifuge tube of known weight, after which 0.8 mL of distilled water was added to each tube. The tubes were vortexed for 5 s until suspended and put on a thermomixer (Thermomixer^®^, Eppendorf AG, Hamburg, Germany) at 1400 rpm for 10 min at 25 °C. The solvent, 0.4 mL isopropanol-lactic acid stock solution was then added to each tube. The flour solvent suspension was again vortexed for 5 s until suspended and put on a thermomixer at 1400 rpm for 10 min at 25 °C. The suspended sample was then centrifuged at exactly 100× *g* for 5 min. The supernatant liquid was discarded and the tube drained. The tube weight was determined and the SIG value calculated as a percentage of flour weight on a 14% moisture basis.

### 4.5. Size Exclusion—High Performance Liquid Chromatography

Proteins from wheat flour were extracted using a two-step procedure [32]. The first step involves the extraction of proteins soluble in an SDS buffer while the second step involves sonication. SE-HPLC analysis was done on the Shimadzu HPLC system equipped with a PDA detector and using a Phenomenox BIOSEP-SEC 4000 column (300 × 4.6 mm). The protein fractions were calculated based on percentage of the respective peak areas relative to the total area using CLASS VP^TM^ software. Step one yielded the SDS extractable fractions and step two the SDS insoluble fractions as four peaks, where fraction 1 = high molecular weight polymeric proteins, fraction 2 = low molecular weight polymeric protein fractions, fraction 3 = gliadins, and fraction 4 = albumins and globulins. Large unextractable polymeric proteins (LUPP) were calculated as the large unextractable fraction as a percentage of the large extractable and unextractable fraction. Total unextractable polymeric proteins (UPP), was the large and small unextractable proteins as a percentage of the total large and small extractable and unextractable fractions.

### 4.6. Statistical Analysis

Analysis of variance was done for trials combined for the two seasons with Agrobase software [31]. The same software was used for correlation analysis between measured characteristics.

## Figures and Tables

**Table 1:** Solvent retention capacity, swelling index of glutenin, SDS sedimentation and flour protein content for six cultivars averaged for two seasons.

Cultivar	Treatment	Distilled Water SRC %	Sodium Carbonate SRC %	Sucrose SRC %	Lactic Acid SRC %	SIG %	SDSS mL	FPC %
Mexicali	Control	79.84	91.88	106.62	109.31	5.18	12.00	10.74
C75	Mod drought	80.85 *	96.72 *	101.94 *	112.59 *	5.15	9.75 *	11.46 *
	Sev drought	76.19 *	83.31 *	96.92 *	105.41 *	5.68 *	11.72	13.73 *
	Mod heat	77.51 *	83.30 *	96.55 *	106.80 *	5.46 *	10.54 *	11.14 *
	Sev heat	78.25 *	86.60 *	101.80 *	119.12 *	6.04 *	12.41	14.14 *
	Mean	78.53	88.36	100.77	110.65	5.50	11.28	12.24
Yavaros	Control	81.36	96.44	106.14	103.39	5.02	9.13	11.00
C79	Mod drought	82.91 *	99.27 *	102.38 *	105.06 *	4.70 *	8.25	12.30 *
	Sev drought	81.50	87.02 *	102.70 *	102.92	5.16 *	8.60	15.71 *
	Mod heat	79.27 *	83.28 *	100.12 *	103.81	5.03	9.25	11.55 *
	Sev heat	81.23	89.31 *	104.63 *	109.64 *	5.34 *	9.41	14.15 *
	Mean	81.25	91.06	103.19	104.96	5.05	8.93	12.94
Altar	Control	79.24	92.97	104.98	102.60	5.36	9.88	10.68
C84	Mod drought	81.64 *	96.69 *	103.12 *	108.94 *	5.14 *	9.38	11.80 *
	Sev drought	79.92 *	84.96 *	100.31 *	103.65	5.66 *	9.38	14.40 *
	Mod heat	78.38 *	82.94 *	98.39 *	104.23 *	5.25 *	9.41	11.38 *
	Sev heat	80.32 *	88.13 *	103.30 *	112.27 *	5.90 *	10.25	13.56 *
	Mean	79.90	89.14	102.02	106.34	5.46	9.66	12.36
Atil	Control	76.06	85.99	98.91	108.97	5.71	14.38	11.78
C2000	Mod drought	75.86	87.05 *	99.40	113.12 *	5.50 *	12.88 *	13.42 *
	Sev drought	74.23 *	81.23 *	96.18 *	106.38 *	5.86 *	13.29 *	16.44 *
	Mod heat	73.05 *	78.79 *	94.18 *	108.58	5.67	14.03	12.52 *
	Sev heat	76.83	87.37 *	102.29 *	115.43 *	6.20 *	12.19 *	15.25 *
	Mean	75.21	84.09	98.19	110.50	5.79	13.35	13.88
Jupare	Control	77.93	89.13	99.44	105.36	5.47	10.75	10.98
C2001	Mod drought	81.53 *	92.21 *	103.76 *	110.13 *	5.57 *	10.00	12.32 *
	Sev drought	77.30	82.55 *	97.85 *	105.58	5.77 *	11.10	15.67 *
	Mod heat	76.12 *	80.27 *	95.24 *	108.20 *	5.68 *	12.16 *	11.96 *
	Sev heat	77.86	88.05 *	102.97 *	113.79 *	6.22 *	10.78	13.52 *
	Mean	78.15	86.44	99.85	108.61	5.74	10.96	12.89
Cirno	Control	73.10	82.73	93.10	94.69	4.92	11.00	10.88
C2008	Mod drought	75.88 *	86.73 *	96.27 *	110.81 *	5.41 *	12.75 *	12.43 *
	Sev drought	75.32 *	80.23 *	95.87 *	101.85 *	5.43 *	11.16	15.85 *
	Mod heat	72.85	74.56 *	91.31 *	102.00 *	5.42 *	12.47 *	11.95 *
	Sev heat	76.31 *	83.11	99.51 *	114.15 *	5.85 *	12.63 *	15.55 *
	Mean	74.69	81.47	95.21	104.70	5.41	12.00	13.33
LSD treatments (*p* ≤ 0.05)		0.61	0.63	0.78	1.14	0.06	1.03	0.15

* significantly different from control value at *p* ≤ 0.05, Mod = moderate, Sev = severe, SRC = solvent retention capacity, SIG = swelling index of glutenin, SDSS = sodium dodecyl sulphate sedimentation, FPC = flour protein content.

**Table 2:** SDS extractable and unextractable gluten proteins (%) combined for two seasons.

		SDS Extractable Proteins				SDS Unextractable Proteins					
Cultivar	Treatment	HMW	LMW	Gliadin	AG	HMW	LMW	Gliadin	AG	LUPP	UPP
Mexicali	Control	3.63	19.37	33.05	8.19	6.51	19.88	4.33	1.01	42.94	61.96
C75	Mod drought	4.96 *	17.02 *	29.05 *	7.55 *	6.04 *	17.35 *	4.94 *	1.01	56.49 *	55.60 *
	Sev drought	4.38 *	16.11 *	43.71 *	6.51 *	4.23 *	23.04 *	3.48 *	0.73 *	51.56 *	49.58 *
	Mod heat	4.78 *	17.14 *	40.93 *	7.45 *	5.36 *	18.88 *	3.41 *	0.62 *	56.33 *	54.69 *
	Sev heat	6.42 *	16.78 *	38.68 *	7.68 *	5.31 *	17.58 *	3.16 *	0.83 *	48.04 *	51.91 *
	Mean	4.83	17.28	37.08	7.48	5.49	19.35	3.86	0.84	51.07	54.75
Yavaros	Control	6.73	19.02	36.97	8.80	6.39	25.30	3.99	0.88	67.60	56.73
C79	Mod drought	5.50 *	19.66	39.52 *	7.78 *	5.94 *	21.39 *	3.04 *	0.84	41.98 *	47.90 *
	Sev drought	5.24 *	20.44 *	39.32 *	6.03 *	6.35	15.99 *	3.03 *	0.62 *	50.24 *	46.29 *
	Mod heat	5.83 *	21.58 *	42.29 *	7.59 *	5.25 *	15.63 *	2.70 *	0.65 *	46.19 *	42.04 *
	Sev heat	5.08 *	20.03 *	45.23 *	7.42 *	4.82 *	15.75 *	2.78 *	0.65 *	53.71 *	45.12 *
	Mean	5.68	20.15	40.67	7.52	5.75	18.81	3.11	0.73	51.94	47.62
Altar	Control	4.81	20.84	37.90	6.47	5.79	21.63	3.11	1.14	54.50	46.49
C84	Mod drought	5.98 *	17.02 *	38.02	7.12 *	5.90	21.11	3.16	0.86 *	45.57 *	45.68
	Sev drought	5.15	18.36 *	39.17	6.36	5.31 *	16.52 *	2.92	0.60 *	54.93	49.34 *
	Mod heat	6.29 *	19.40 *	41.84 *	7.47 *	5.24 *	13.22 *	2.66 *	0.52 *	43.67 *	42.90 *
	Sev heat	5.64 *	17.38 *	42.85 *	6.15	4.85 *	18.24 *	3.66 *	0.74 *	44.70 *	45.55
	Mean	5.57	18.60	39.96	6.71	5.42	18.14	3.10	0.77	48.67	45.99
Atil	Control	4.79	18.53	46.09	7.17	5.24	15.01	2.98	0.63	50.71	45.94
C2000	Mod drought	5.38 *	16.48 *	46.68	6.71 *	4.92	23.30 *	3.38 *	1.37 *	47.52 *	50.94 *
	Sev drought	5.29 *	17.25 *	50.52 *	6.32 *	7.10 *	16.33 *	4.02 *	0.68	55.97 *	53.08 *
	Mod heat	7.72 *	17.48 *	50.27 *	7.99 *	7.70 *	20.01 *	2.00 *	0.69	40.87 *	58.59 *
	Sev heat	5.05	17.37 *	55.51 *	6.84	4.38 *	16.01 *	4.00 *	0.55	40.65 *	44.31
	Mean	5.65 *	17.42 *	49.81 *	7.01	5.87 *	18.13	3.28	0.78	47.14 *	50.57 *
Jupare	Control	4.86	18.26	40.09	6.92	4.10	20.84	2.58	1.63	50.45	51.12
C2001	Mod drought	6.80 *	17.05 *	41.58	6.57	5.32	19.78 *	3.28 *	0.70 *	56.93 *	55.70 *
	Sev drought	5.21	17.10 *	45.35 *	6.98	5.54	15.26 *	2.30	0.81 *	59.97 *	43.27 *
	Mod heat	4.70	21.13 *	32.91 *	6.98	4.96	18.33 *	2.55	0.53 *	66.04 *	45.72 *
	Sev heat	7.01 *	17.55	44.25 *	5.82 *	4.25	16.31 *	2.32	0.67 *	43.69 *	43.68 *
	Mean	5.72	18.22	40.84	6.65	4.83	18.10	2.61	0.87	55.42	47.90
Cirno	Control	3.35	17.79	41.87	6.30	5.78	20.93	3.15	0.72	46.22	52.52
C2008	Mod drought	6.12 *	19.27 *	42.73	6.34	4.64	16.17 *	2.65 *	0.88	42.99 *	44.83 *
	Sev drought	5.25 *	17.79	42.74	7.91 *	6.46	14.62 *	3.04	0.58	52.16 *	56.53 *
	Mod heat	5.31 *	15.87 *	43.92 *	8.08 *	5.31	16.43 *	3.28	0.57	50.32 *	45.51 *
	Sev heat	7.43 *	15.67 *	41.59	5.52 *	6.04	18.79 *	2.35 *	0.65	47.69	42.54 *
	Mean	5.49	17.28	42.57	6.83	5.65	17.39	2.89	0.68	47.88	48.39
LSD treatment (*p* ≤ 0.05)		0.38	0.90	1.59	0.37	0.37	1.00	0.33	0.18	2.49	2.40

* significantly different from control value at *p* ≤ 0.05, Mod = moderate, Sev = severe, HMW = high molecular weight, LMW = low molecular weight, AG = albumin/globulins, LUPP = large unextractable polymeric proteins, UPP = total unextractable polymeric proteins.

**Table 3:** Solvent retention capacity, swelling index of glutenin, SDS sedimentation and flour protein content averaged for six cultivars and two seasons.

Treatment	Distilled Water SRC %	Sodium Carbonate SRC %	Sucrose SRC %	Lactic Acid SRC %	SIG %	SDSS mL	FPC %
Control	77.92 (3)	89.86 (2)	6.8 (1)	104.05 (5)	5.33 (3)	11.19 (3)	11.01 (5)
Mod drought	79.78 * (1)	93.11 * (1)	4.0 * (3)	110.11 * (2)	5.24 * (5)	10.50 * (5)	12.29 * (3)
Sev drought	77.41 (4)	83.21 * (4)	4.6 * (2)	104.30 (4)	5.59 * (2)	10.87 * (4)	15.30 * (1)
Mod heat	76.20 * (5)	80.52 * (5)	3.5 * (5)	105.60 * (3)	5.25 * (4)	11.31 (1)	11.75 * (4)
Sev heat	78.47 (2)	87.09 * (3)	3.8 * (4)	114.07 * (1)	5.92 * (1)	11.28 (2)	14.36 * (2)
LSD (0.05)	0.61	0.63	0.78	1.14	0.06	0.23	0.15

* significantly different from control value at *p* ≤ 0.05, ranking in parentheses. Mod = moderate, Sev = severe, SRC = solvent retention capacity, SIG = swelling index of glutenin, SDSS = sodium dodecyl sulphate sedimentation, FPC = flour protein content.

**Table 4:** SDS extractable and unextractable gluten protein fractions (%) averaged for six cultivars and two seasons.

	SDS Extractable Proteins				SDS Unextractable Proteins					
	HMW	LMW	Gliadin	AG	HMW	LMW	Gliadin	AG	LUPP	UPP
Control	4.70 (5)	18.97 (1)	39.33 (5)	7.31 (2)	5.63 (2)	20.6 (1)	3.35 (3)	1.00 (1)	52.07 (2)	52.46 (1)
Mod heat	5.77 * (3)	18.77 (2)	43.47 * (2)	7.59 * (1)	5.63 (3)	17.08 * (4)	2.76 (5)	0.60 * (5)	50.57 (3)	48.24 * (4)
Sev heat	6.10 * (1)	17.46 * (5)	44.68 * (1)	6.57 * (5)	4.94 * (5)	17.11 * (3)	3.04 (4)	0.68 (3)	46.41 * (5)	45.52 * (5)
Mod drought	5.79 * (2)	17.75 * (4)	39.60 (4)	7.01 (3)	5.46 (4)	19.85 (2)	3.41 (1)	0.94 (2)	48.58 * (4)	50.11 (2)
Sev drought	5.09 *(4)	17.84 * (3)	42.03 * (3)	6.69 * (4)	5.83 (1)	16.96 * (5)	3.13 (2)	0.67 * (4)	54.14 (1)	49.68 * (3)
LSD (treat)	0.38	0.90	1.59	0.37	0.37	1.00	0.83	0.33	2.49	2.40

* significantly different from control value at *p* ≤ 0.05, ranking in parentheses. Mod = moderate, Sev = severe, HMW = high molecular weight, LMW = low molecular weight, AG = albumin/globulins, LUPP = large unextractable polymeric proteins, UPP = total unextractable polymeric proteins.

**Table 5:** Significant correlations between solvent retention capacity, protein fractions, flour protein content and SDS sedimentation under different stress treatments.

Optimal			Moderate Drought			Severe Drought		
Distilled water SRC	LUPP	0.54 **	Sodium carobonate SRC	ExGli	−0.62 **	Distilled water SRC	ExHMW	−0.71 **
Distilled water SRC	UPP	0.49 *	Sodium carobonate SRC	ExAG	0.52 *	Distilled water SRC	SDSS	−0.56 **
Lactic acid SRC	FPC	0.76 **	Sodium carobonate SRC	UnHMW	0.51 *	Sodium carobonate SRC	ExHMW	−0.76 **
Sodium carobonate SRC	FPC	0.48 *	Sodium carobonate SRC	FPC	−0.56 **	Sodium carobonate SRC	SDSS	−0.49 *
SIG	UnLMW	−0.58 **	Sodium carobonate SRC	SDSS	−0.79 **	Sucrose SRC	SDSS	−0.64 **
SIG	SDSS	0.67 **	Sucrose SRC	ExGli	−0.44 *	Sucrose SRC	ExHMW	−0.66 **
SIG	FPC	0.51 *	Sucrose SRC	SDSS	−0.60 **	Lactic acid SRC	ExHMW	−0.78 **
			Lactic acid SRC	SDSS	0.69 **	SIG	SDSS	0.70 **
			Lactic acid SRC	LUPP	0.44 *	SIG	ExLMW	−0.50 *
			Distilled water SRC	SDSS	−0.68 **			
			SIG	SDSS	0.60 **			
			SIG	ExLMW	−0.46 *			
**Moderate Heat**			**Severe Heat**					
Lactic acid SRC	FPC	0.46 *	Sucrose SRC	ExLMW	0.65 **			
Distilled water SRC	FPC	−0.49 *	Sucrose SRC	UnLMW	−0.71 **			
Distilled water SRC	ExGli	−0.45 *	SIG	ExAG	−0.53 *			
Distilled water SRC	ExLMW	0.47 *	Distilled water SRC	ExLMW	0.60 **			
Sucrose SRC	SDSS	−0.56 **	Distilled water SRC	UnLMW	−0.53 *			
Distilled water SRC	SDSS	−0.67 **	Lactic acid SRC	SDSS	0.68 **			
Sodium SRC	SDSS	−0.52 *	Distilled water SRC	SDSS	−0.64 **			
SIG	SDSS	0.86 ***	Sodium SRC	SDSS	−0.46 *			
SIG	FPC	0.62 **						

* *p* ≤ 0.05, ** *p* ≤ 0.01, *** *p* ≤ 0.001, SRC = solvent retention capacity, SIG = swelling index of glutenin, SDSS = sodium dodecyl sulphate sedimentation, FPC = flour protein content, LUPP = large unextractable polymeric proteins, UPP = total unextractable polymeric proteins, Un = SDS unextractble, Ex = SDS extractable, LMW = low molecular weigh glutenin, Gli = gliadin, HMW = high molecular weight glutenin.

**Table 6:** List of genotypes with their high- and low molecular weight glutenin subunit composition as determined by SDS-polyacrylamide gel electrophoresis.

Genotypes	HMW-GS	LWM-GS		
	*Glu-B1*	*Glu-A3*	*Glu-B2*	*Glu-B3*
MexicaliC75	7 + 8	6	12	2 + 4 + 15 + 19
YavarosC79	7 + 8	6	12	2 + 4 + 15 + 19
AltarC84	7 + 8	6	12	2 + 4 + 15 + 19
AtilC2000	7 + 8	6	12	2 + 4 + 15 + 19
JupareC2001	7 + 8	6	12	2 + 4 + 15 + 19
CirnoC2008	7 + 8	6	12	2 + 4 + 15 + 19

HMW-GS = high molecular weight-glutenin subunits, LMW-GS = low molecular weight-glutenin subunits.

**Table 7:** Temperature data (°C) in Obregon, Mexico, season 1 and 2.

Maximum Temp Season 1	Average Temp Season 1	Minimum Temp Season 1	Month	Maximum Temp Season 2	Average Temp Season 2	Minimum Temp Season 2
28.5	19.8	13.8	Nov.	27.8	20.5	14.7
25.6	16.7	9.9	Dec.	25.4	16.6	9.8
23.6	14.1	6.6	Jan.	26.9	16.5	8.5
23.8	14.1	6.0	Feb.	27.0	17.0	9.5
29.1	18.5	9.5	Mar.	28.6	18.9	11.0
31.1	20.6	11.0	Apr.	32.2	21.5	11.8
35.3	25.3	15.0	May	36.5	25.9	15.1
36.7	28.9	21.5	Jun.	38.7	31.4	24.8

**Table 8:** Chemical and physical properties for the soil profile at Ciudad Obregon, Mexico [28,29].

Depth (cm)	OM (%)	Total N (%)	pH	P Olsen (ppm)	CEC (Meq/100 g)	Particle Size Fraction (%)			BD g/cm^3^
						Sand	Silt	Clay	
0–15	1.2	0.04	8.9	17	33.9	32	18	50	1.27
15–40	0.9	0.06	8.9	6	30.5	34	18	48	1.20
40–70	0.7	0.03	8.4	3	32.4	32	16	52	1.38
70–120	0.3	0.01	5.9	3	18.7	24	16	60	1.37

OM = organic matter, N = nitrogen, CEC = cation exchange capacity, BD = bulk density.

## Data Availability

Data is available from the authors.
